# Comparing the Above and Below-Ground Chemical Defences of Three *Rumex* Species Between Their Native and Introduced Provenances

**DOI:** 10.1007/s10886-023-01427-0

**Published:** 2023-05-01

**Authors:** Cristian-Andrei Costan, William Godsoe, Jennifer L. Bufford, Philip E. Hulme

**Affiliations:** 1 Bio-Protection Research Centre, Lincoln, Canterbury 7647 New Zealand; 2 Foundation for Arable Research, Templeton, Canterbury 7678 New Zealand; 3 Manaaki Whenua – Landcare Research, Lincoln, Canterbury 7647 New Zealand

**Keywords:** Alien species, Native, Introduced, Chemical defences, Plant invasion, Weeds

## Abstract

Compared to their native range, non-native plants often experience reduced levels of herbivory in the introduced range. This may result in reduced pressure to produce chemical defences that act against herbivores. We measured the most abundant secondary metabolites found in *Rumex* spp., namely oxalates, phenols and tannins. To test this hypothesis, we compared native (UK) and introduced (NZ) provenances of three different *Rumex* species (*R. obtusifolius, R. crispus* and *R. conglomeratus*, Polygonaceae) to assess whether any significant differences existed in their levels of chemical defences in either leaves and roots. All three species have previously been shown to support a lower diversity of insect herbivores and experience less herbivory in the introduced range. We further examined leaf herbivory on plants from both provenances when grown together in a common garden experiment in New Zealand to test whether any differences in damage might be consistent with variation in the quantity of chemical defences. We found that two *Rumex* species (*R. obtusifolius* and *R. crispus*) showed no evidence for a reduction in chemical defences, while a third (*R. conglomeratus*) showed only limited evidence. The common garden experiment revealed that the leaves analysed had low levels of herbivory (~ 0.5%) with no differences in damage between provenances for any of the three study species. Roots tended to have a higher concentration of tannins than shoots, but again showed no difference between the provenances. As such, the findings of this study provide no evidence for lower plant investments in chemical defences, suggesting that other factors explain the success of *Rumex* spp. in New Zealand.

## Introduction

In order to defend themselves against herbivory, plants produce secondary metabolites that function as chemical defences. Secondary metabolites can help with plant growth and development, but are not essential for survival (Gols 2014; Erb and Kliebenstein 2020). Non-native plants frequently escape herbivores from the native range and often experience reduced herbivory in the introduced range (Joshi and Vrieling 2005; Müller-Schärer et al. 2004; Costan et al. 2022). Secondary metabolites such as oxalates, phenols and tannins act against both invertebrate and vertebrate herbivores and occur in high concentrations (Poorter and Jong 1999; Peschiutta et al. 2020; Damestoy et al. 2019). Since herbivore pressure is often reduced in the introduced range, it is possible that plants could decrease their production of such chemical defences. This could result in saved resources which can be reallocated towards growth and reproduction, giving introduced plants a competitive advantage over local plant species (Müller-Schärer et al. 2004). Most studies looking at plant chemical defences of non-native plant species do not compare chemical defences in above vs. belowground tissues (van Dam 2009), although both roots and shoots often contain defence phytochemicals (Rasmann and Agrawal 2008; Johnson et al. 2016).

*Rumex* species in New Zealand represent a suitable test case to examine whether reduced herbivore pressure in the introduced range results in a reduction in secondary metabolites. In the native range, *Rumex* plants are associated with a range of invertebrate herbivores (Salt and Whittaker 1998), including the specialist shoot herbivore *Gastrophysa viridula* (De Geer, 1775) and the root herbivore *Pyropteron chrysidiforme* (Esper, 1782). A comparative study of insect herbivores associated with three *Rumex* species in their native UK range and their introduced range in New Zealand found an absence of specialist herbivores in the latter region as well as a lower diversity of phytophagous insects (Costan et al. 2022). Furthermore, this study also demonstrated that *Rumex* plants (in particular *R. crispus*) experienced a sevenfold lower level of herbivore damage in New Zealand (Costan et al. 2022). Given this shift in herbivore pressure, *Rumex* species would appear good candidates for having evolved to redirect resources towards growth and reproduction instead of the production of chemical defences.

*Rumex* species are known for abundant chemical defences. The most abundant defensive compound in *Rumex* plants is the organic compound oxalate, a secondary metabolite comprising of up to 90% of the total ionic metabolites (Miyagi et al. 2010). When oxalates are consumed by herbivores they form an insoluble salt, calcium oxalate, which can lead to the formation of renal crystals in both vertebrates and invertebrates (Reynolds et al. 2021). Phenolics may also function as defence against herbivores due to their protein-binding (Haslam 1988) and oxidative (Salminen et al. 2011) capacity. Among the wide array of chemical defences in *Rumex*, tannins are especially toxic (Demirezer et al. 2001). A subgroup of phenolics, tannins are commonly found in both the shoots and roots of plants and form insoluble complexes with dietary protein that reduce digestive capacity in herbivorous insects (Yuan et al. 2020). However, for most insects (lepidopteran larvae with highly alkaline gut conditions in particular), the potential pro-oxidative activity of tannins (ellagitannins) has a higher biological importance than protein-precipitating effects (Appel 1993; Salminen and Karonen 2011).

In this study, we measured the levels of the most abundant chemical defences (i.e. oxalates, phenols and tannins) in three *Rumex* species (*R. conglomeratus, R. crispus and R. obtusifolius*) from both the above- (the entirety of the plant that wasn’t growing in the soil substrate) and below-ground (the entire root system) parts of the plants. All three *Rumex* species are short-lived perennial herbaceous species native to Eurasia and occupy a wide range of natural and cultivated habitats (Kubát 1990). Since all three species were introduced to New Zealand over 150 years ago (Webb et al. 1988) and have experienced a reduction in herbivore pressure following their introduction to New Zealand (Costan et al. 2022), there has been ample opportunity for rapid evolution (Maron et al. 2004). We tested for a reduction in herbivore defences in *Rumex* by conducting analyses of secondary metabolites in the above and below ground tissue of *Rumex* plants from native and introduced ranges grown under similar environmental conditions. Furthermore, we extended these laboratory analyses to examine herbivory on plants from the native and introduced provenances grown together in common garden experiments under natural conditions in the field in New Zealand. The common garden study aimed to test whether any differences in damage might be consistent with variation in the quantity of chemical defenses.

## Methods

### Plant Sample Preparation

The plant material used in the chemical analysis was collected from plants grown under similar environmental conditions in the greenhouses of Lincoln University, New Zealand. For each species, four plants were grown from seeds collected from each of four different regions in each provenance: east England, southwest England, southwest Scotland and southeast Scotland from the United Kingdom (UK, native range) and Canterbury, Westland, Otago and Southland from New Zealand (NZ, introduced range). Thus, a total of 32 plants for each study species were used. The plants were grown in individual pots under the same glasshouse environmental conditions in New Zealand and harvested 60 days after transplant. The harvesting date was chosen because the early life stage of plants is crucial for competitive success (Weaver and Cavers 1979) and it ensured that plants would not become pot bound, which could have altered the levels of secondary metabolites in the plants (Baldwin 1988). Above and below ground parts of the plants were separated, washed, dried to a constant weight at 65 °C for 48 h and subsequently ground into a fine powder using an electric grinder and stored in sealed plastic containers. Drying plant material before analysis is consistent with other studies (Savage et al. 2000), and has been shown to have minimal impacts on total phenol or tannin concentrations (Harbourne et al. 2009). Furthermore, since all plant samples were dried together, drying is unlikely to affect the comparison of secondary chemicals between provenances.

### Oxalic Acid Analysis

Oxalic acid measurements used the protocol described by Savage et al. (2000). Both soluble and total oxalic acid were extracted from each plant. To extract oxalic acid, between 0.400 and 0.405 g of the ground plant material was measured and mixed with 40 mL distilled water (soluble oxalic acid) or 40 mL of 2 M HCl (total oxalic acid). The samples were then placed in a water bath at 80 °C for 20 min, cooled, and made up to a volume of 100 mL with distilled water for the soluble oxalic acid analysis (Barnstead Nanopure II) or 2MHCl for the total oxalic acid analysis. Extracts were centrifuged at 3000 rpm and 10 mL of the supernatant was filtered through a 0.45 mm cellulose acetate membrane (Satorius, Goettingen, Germany). From this, a sample of 5 µL was analysed using a Waters Chromatography System, consisting of a Waters 717 Plus auto-sampler, a Waters 600-MS Isocratic/Gradient Pump and a Waters UV/VIS detector set at 210 nm. Millennium (ver 2.15) chromatographic software was used to process the output. Chromatographic separation was conducted using an Aminex Ion exclusion HPX-87 H 300 × 7.8 mm analytical column attached to an Aminex Cation-H guard column, using an isocratic elution at 0.5 mL/min with 0.0125 M sulphuric acid (Analar, BDH, UK) as a mobile phase. Before use and between sample sets, the analytical column was held at room temperature and the columns were equilibrated at a flow rate of 0.1 mL/min. Prior to use, the mobile phase was filtered through a 0.45 μm membrane, followed by degassing using a vacuum. The total oxalic acid peak was identified by comparing the retention time to standard curves in the range of 1 to 20 mg/100 mL. The standard curves were prepared by diluting oxalic acid (Analar, BDH, UK) with distilled water (soluble oxalic acid) or 2.0 M HCl (total oxalic acid). Prior to analysis, all blank and standard solutions were filtered through a 0.45 μm cellulose acetate membrane syringe filter.

### Total Phenolics Determination

Total phenolics and oxidative capacity measurements were carried out using the protocol outlined by Salminen and Karonen (2011). For the assessment of total phenolics and oxidative capacity, 10 ± 0.5 mg of ground plant material was extracted three times over a period of 2 h with 800 µL of acetone/water (7/3, v/v) and vortexed for 5 min. After each extraction step, the supernatant of the solutions was separated by centrifugation in an Eppendorf centrifuge (10 min at 14.000 rpm) and decanted into a new 2 ml Eppendorf tube. The samples were then placed into an Eppendorf concentrator, and the acetone was evaporated. Aqueous samples were frozen at -20 °C and lyophilized. The freeze-dried phenolic extract was re-suspended in 500 µL of milipure water, vortexed for 5 min and centrifuged for 10 min at 14.000 rpm. The supernatant was then filtered through a 0.45 mm cellulose acetate membrane (Satorius, Goettingen, Germany) and 20 µL of the solution was mixed with 280 µL of a buffer (9/5 v/v 10 pH carbonate buffer/0.6% formic acid) and vortexed for 5 min. Using a 96 well plate reader, 50 µL of this mixture was moved into a single plate reader well, to which 50 µL of 1 N Folin-Cicolteau reagent and 100 µl 20% sodium carbonate solution were added. The plate was incubated at 25 °C (shaken for 10 s every 10 min) for 60 min and the absorbance was read at 730 nm. All samples were analysed in three replicates. Five samples from *R. conglomeratus* were outliers in their initial phenolic concentrations, so phenols were re-measured for these samples and the second, less extreme, value was used. In order to obtain a standard curve, the protocol described above was used with a series of gallic acid dilutions (0.1, 0.5, 1.0, 1.5 and 2.0 mg/mL), which were made by dissolving the gallic acid in a volumetric flask with a few drops of ethanol and diluted with purified water.

### Ellagitannins Determination

To determine the contribution of ellagitannins (easily oxidized tannins) to the total phenolic concentration we oxidized a sample of the extract used to test for total phenols. Samples were diluted or concentrated as needed until total phenolic concentration was 1.0 ± 10% to ensure comparable results. To oxidize the phenolics in the extract, 20 µL of extract was placed into a single well of a 96 well plate reader and 180 µL of 10 pH carbonate buffer was added. The plate was then incubated at 25 °C (shaken for 10 s every 10 min) for 90 min, after which 100 µL of 0.6% formic acid was added, changing the sample pH from 10 to 6 and stopping oxidation. From these oxidized mixtures 50 µL was placed into a new single well of a 96 well plate reader, on top of which 50 µL of 1 N Folin-Cicolteau reagent and 100 µl of 20% sodium carbonate solution were added. The plate was incubated at 25 °C (shaken for 10 s every 10 min) for 60 min and the absorbance was read at 730 nm. Three replicates of each sample were analysed.

### Total Tannins Determination

The total tannin measurements were carried out using the radial diffusion protocol described by Hagerman (1987). A 1% (w/v) solution of agarose was prepared in a buffer (50 mM acetic acid and 60 µM ascorbic acid, adjusted to a pH of 5.0) by heating the suspension of agarose to boiling while stirring. The solution was then allowed to cool down in a water bath set at 45 °C, after which 0.1% w/v BSA protein was slowly added, while gently stirring. After homogenization, the solution was dispersed in 9.5 ml aliquots into standard Petri dishes (8.5 cm diameter) set on a flat surface and allowed to cool. Once the agarose plates reached room temperature they were sealed with Parafilm and stored at 4 °C to prevent bacterial growth.

To assess total tannins, 100 ± 0.5 mg of the ground plant material from each sample was vortexed for 5 min. The plant material was extracted at room temperature for half an hour, using 0.5 mL of 50% (v/v) aqueous methanol, after which it was centrifuged for 10 min at 14,000 rpm. The tannin containing solutions were added to four equally spaced 4.0 mm wells punched in each agarose plate. Six successive aliquots of 8 µL were added to each well, as the solution was absorbed by the agar gel. After placing the samples in the gel, the Petri dishes were covered, sealed with Parafilm and incubated at 30 °C for 120 h, after which the diameters of the rings were measured. For each ring two measurements at right angles to each other were taken and averaged, in order to minimize errors due to non-uniform ring development. In order to obtain a standard curve, the same protocol was used with a series of tannic acid dilutions (0.2, 0.4, 0.6, 0.8, 1.0, 2.0, 3.0 and 4.0 mg/mL), which were made by dissolving tannic acid in purified 50% (v/v) aqueous methanol.

### Herbivory in Common Gardens

For each *Rumex* species, seeds from six regions, three sampled in the UK and NZ each, were planted in two common field gardens using a randomized complete block design. The seeds used were sourced from a subset of the same regions, and often included the same parent plants, as those seeds used for the greenhouse experiment. Field gardens were established in the New Zealand regions of Westland and Southland (Bufford and Hulme 2021). These sites represent a very wet (Westland, 2,384 mm/year, 6.9 °C mean minimum) and a moderately dry, cold (Southland, 1,156 mm/year, 5.3 °C mean minimum) environment. Plants were spaced at least 0.5 m apart and the experiments were surrounded by fencing to exclude large vertebrate herbivores. After approximately one year of growth, the largest leaf from each of approximately 90 plants per provenance (UK vs. NZ) was photographed in November (austral spring) to determine the percent of leaf area damaged by herbivores. The collected leaves were analysed using the image processing program BioLeaf, according to the protocols described by Machado et al. (2016) and Costan et al. (2022).

### Statistical Analysis

Linear mixed effects models (LMMs) were used to examine variation between provenances in oxalic acid (mg/g dry matter), phenols (mg/100 g Gallic Acid Equivalents), ellagitannins (mg/100 g Gallic Acid Equivalents) and tannins (mg/g Tannic Acid Equivalents) for each *Rumex* species. Each model had provenance (UK or NZ) as a fixed effect. We treated provenance as a fixed effect because variables with < 5 levels tend to produce anomalous estimates when treated as random effects in a mixed effects model (Harrison et al. 2018). Region (n = 8) was treated as a random effect. In each model the data were collected from 32 individual plants from each provenance. We used residual plots to determine if transformations were needed. For the analysis of oxalic acid content, the data were log-transformed; no transformation was needed for analyses of the other chemicals. The analysis of the percentage leaf damage in the common garden experiments used LMMs with provenance (UK or NZ) and site as fixed effects and with region and population as random effects. Leaf damage was calculated as an average across 30 leaves per provenance per species, and therefore the residuals indicated that no transformation was required. The analyses of each *Rumex* species were run separately.

We assessed the significance of the provenance effect with a Wald chi-squared test for all models. Residual plots and diagnostics were checked to ensure the validity of the model assumptions. All statistical analyses were performed in R (R Core Team, 2019). LMMs and GLMMs were run using the ‘lme4’ R package v.1.1–19 (Bates et al. 2015).

## Results

**Fig. 1:**
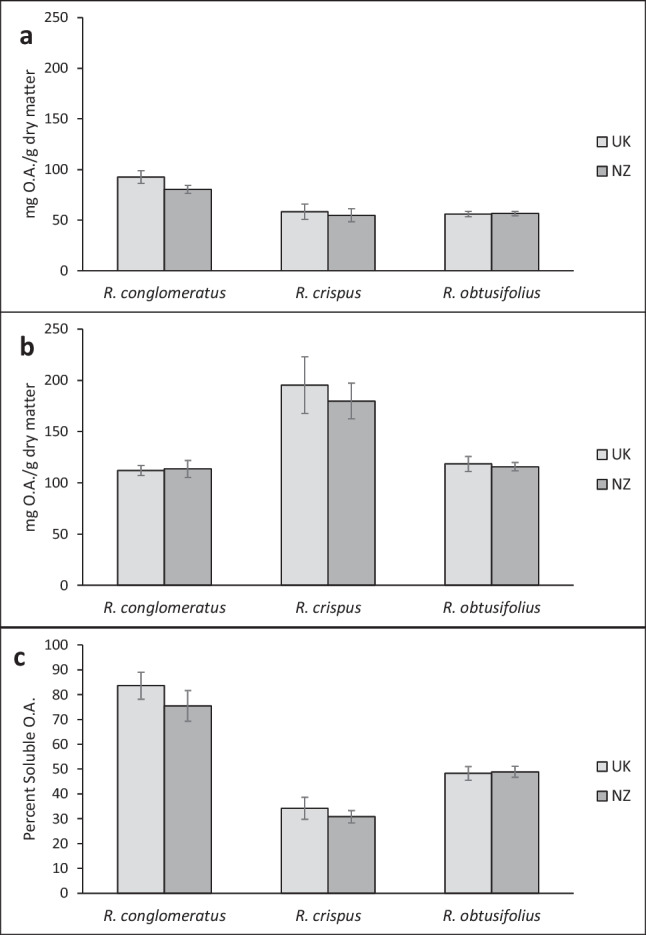
Leaf soluble (**a**), total (**b**) and percent soluble (**c**) oxalic acid content of *Rumex* spp. grown from seeds from the native (United Kingdom, UK) or introduced (New Zealand, NZ) ranges under greenhouse conditions. For each species, 16 replicates per provenance (UK and NZ) were averaged. *Error bars* ± 1SE

**Fig. 2:**
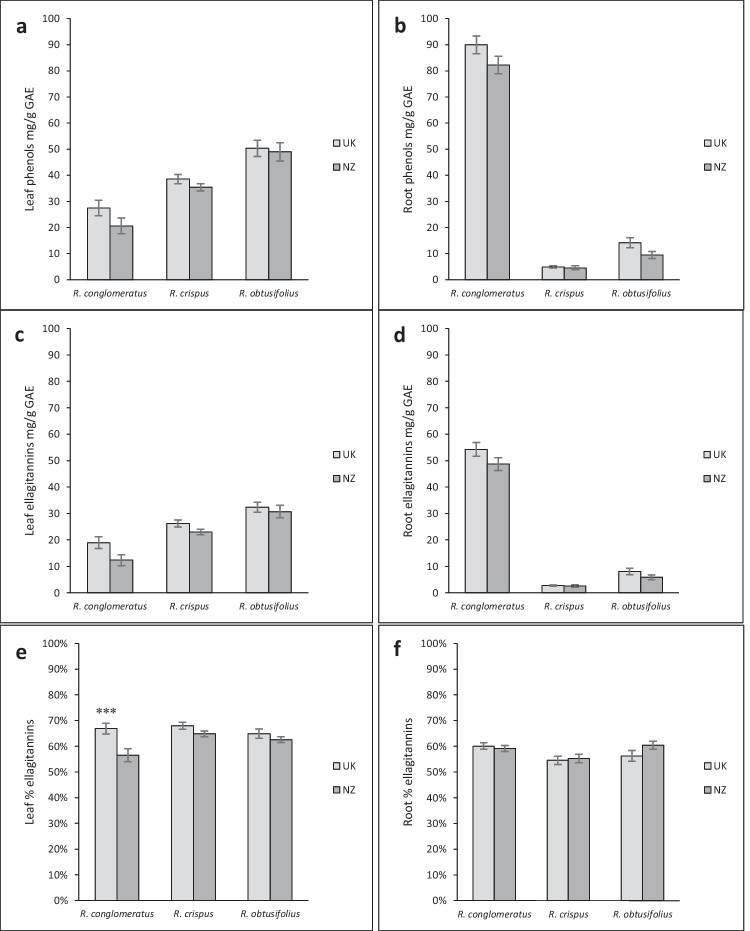
Leaf and roof total phenols (**a** & **b**), ellagitannins (**c** & **d**) and percentage of ellagitannins (**e** & **f**) out of total phenols of *Rumex* spp. grown from seeds from the native (United Kingdom, UK) or introduced (New Zealand, NZ) ranges under controlled greenhouse conditions expressed in GAE (Gallic Acid Equivalent). For each species, 16 replicates per provenance (UK and NZ) were averaged. *Error bars* ± 1SE

**Fig. 3:**
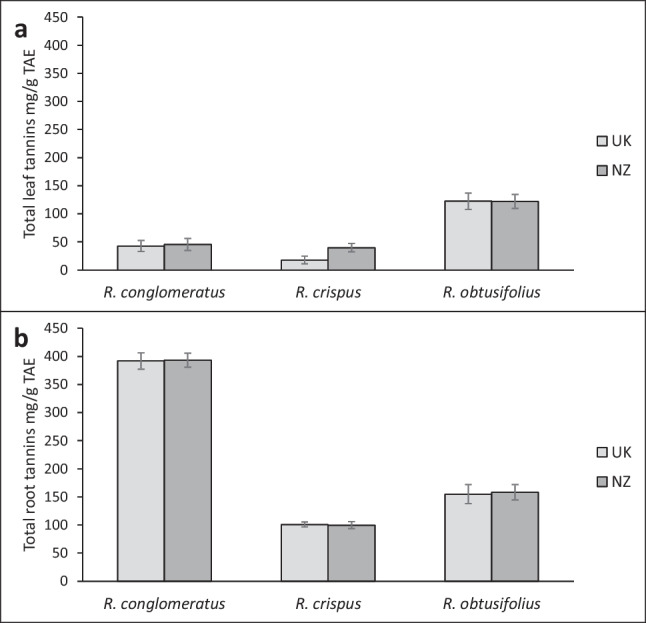
Total leaf (**a**) and root (**b**) tannin content of *Rumex* spp. grown from seeds from the native (United Kingdom, UK) or introduced (New Zealand, NZ) ranges under controlled greenhouse conditions. For each species, 16 replicates per provenance (UK and NZ) were averaged. *Error bars* ± 1SE

**Fig. 4:**
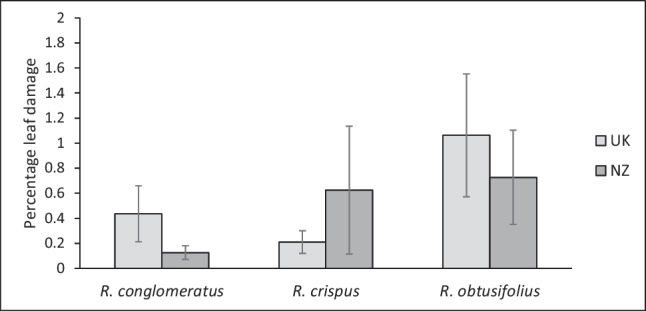
Percentage leaf area damaged by herbivores of plants from the native (UK) and introduced (NZ) provenance when grown together  in common garden experiments in New Zealand. *Error bars* ± 1SE

In most comparisons of plant chemistry there was no statistically significant difference between native and introduced provenances (Table 1). The most notable differences for chemicals were between species (Figs. 1, 2 and 3) rather than between provenances. Herbivory damage did not differ between provenances (Fig. 4).

### Oxalic Acid

There were no statistically significant differences in soluble, total or the percentage of soluble oxalic acid between the leaves of *Rumex* plants from the native (UK) and introduced (NZ) provenances (Table 1). This was true for all three study species (Fig. 1). Oxalic acid in the roots was undetectable in all cases.

### Phenols & Ellagitannins

Native provenance *R. conglomeratus* plants had a significantly higher percentage of ellagitannins in their leaves than introduced provenance plants (p < 0.001), but no differences between provenances were detected for *R. crispus* and *R. obtusifolius* (Table 1). The highest concentration of leaf phenols and ellagitannins was found for *R. obtusifolius*, followed by *R. crispus* and *R. conglomeratus*. All three species showed a strong correlation between the concentration of total phenols and ellagitannins and had roughly the same percentage ellagitannins (Fig. 2).

There were no statistical differences between the native and introduced provenances in regards to the concentrations of total phenols and ellagitannins in the roots of plants (Fig. 2b,d). The concentration of total phenols and ellagitannins was much greater in the roots of *R. conglomeratus* compared to the leaves, while for *R. obtusifolius* and *R. crispus*, total phenols and ellagitannins in the roots were very low. Both leaves and roots of all three *Rumex* study species had approximately 60% ellagitannins (Fig. 2e, f).

### Tannins

No statistically significant differences between provenances were observed for any of the three *Rumex* species in total leaf tannins (Table 1). Two species, *R. conglomeratus* and *R. crispus* had around 40 mg/g Tannic Acid Equivalents (TAE), but *R. obtusifolius* values were over 120 mg/g TAE (Fig. 3). The total tannins in the roots was much higher than total tannins in leaves for all study species (Fig. 3a versus b), with *R. conglomeratus* having around 390 mg/g TAE, *R. crispus* around 100 mg/g TAE and *R. obtusifolius* 155 mg/g TAE. For *R. conglomeratus*, total tannins in the roots were eight times higher than in the leaves (Fig. 3).

### Leaf Herbivory

There were no differences in leaf herbivore damage between plants from the native (UK) and introduced (NZ) provenances when grown in common gardens in New Zealand. The leaf area damaged by herbivory was on average ~ 0.5% (Fig. 4; Table 1).

**Table 1:** Table of the significance of provenance in models of chemical defences and herbivore damage in *Rumex conglomeratus*, *Rumex crispus* and *Rumex obtusifolius*

		*R. conglomeratus*		*R. crispus*		*R. obtusifolius*	
Response variable	Organ	Coefficient estimate	P value	Coefficient estimate	P value	Coefficient estimate	P value
Soluble oxalic acid	Leaf	1.23	0.1	0.37	0.70	-0.05	0.91
Total oxalic acid	Leaf	-0.15	0.89	1.55	0.64	0.27	0.75
Percentage soluble oxalic acid	Leaf	8.25	0.34	3.44	0.50	-0.50	0.91
Total phenols	Leaf	6.90	0.24	3.16	0.37	1.37	0.77
	Root	7.74	0.12	0.34	0.73	4.68	0.20
Ellagitannins	Leaf	6.60	0.14	3.24	0.15	1.62	0.59
	Root	7.74	0.12	0.34	0.73	4.68	0.20
Percentage ellagitannins	Leaf	10.50	**0.01**	3.13	0.12	2.50	0.24
	Root	7.74	0.12	0.34	0.73	4.68	0.20
Total tannins	Leaf	-0.28	0.89	-2.18	0.11	0.052	0.98
	Root	-0.15	0.94	0.12	0.90	-0.32	0.92
Percentage leaf damage	Leaf	0.22	0.68	-0.84	0.13	-0.03	0.93

Coefficients show the difference in introduced provenance (New Zealand) plants compared to native provenance (United Kingdom) plants. *P*-values are calculated from a Wald chi-squared test with one degree of freedom for the fixed effect of provenance. Statistically significant *P*-values are highlighted in bold.

## Discussion

Our work has shown that defensive chemicals in several species of *Rumex* are unchanged in their invasive range. This finding is surprising in view of the shifts in herbivore communities between their native and introduced range. For all three *Rumex* study species, the majority of the chemical defences analysed showed no significant differences between provenances. The one exception was that the percentage ellagitannins in the leaves of *Rumex conglomeratus *from the introduced provenance was significantly lower than that of their counterparts from the native provenance. Leaf herbivory in the common garden experiments was similar for plants from the native and introduced provenances for all three species.

Though it has long been thought that plants in introduced ranges will reduce their defences, our results corroborate other studies which found no clear decrease in secondary metabolites in a species’ introduced ranges (Dooduin and Vrieling 2011). For example, a recent study found that only one of three *Acacia* species (*A. longifolia*) from the introduced provenance showed evidence for a reduction in chemical defences, but there were no corresponding data on levels of herbivory (Manea et al. 2019). In our study, no consistent support was found for a decrease in secondary metabolites in New Zealand. Neither the chemical defences measured, nor the responses of herbivores to plants in field conditions indicated a change in effective defence, implying that both constitutive and induced defences, which would be active under field conditions, are the same between provenances. This is despite the fact that all three *Rumex* species experience less leaf and root damage in the introduced range (Costan et al. 2022).

It is important to keep in mind the timescale of evolution (Bezemer et al. 2014; Chun et al. 2010; Harvey et al. 2010; Santangelo et al. 2022). Previous work suggests that evolution in invasive plants can be surprisingly rapid, occurring within a hundred years for herbaceous species (Colautti and Barrett 2013, Maron et al. 2004; Sun and Roderick 2019). The *Rumex* species considered here set seed within one to two years, yielding an estimate of at least 70 generations since their first introduction to New Zealand 150 years ago (Webb et al. 1988), with most seeds germinating with in the first year or two under good conditions (J Bufford, pers. obs.), providing abundant scope for evolution to occur. However, the strength of any enemy release is expected to decrease over time, as native or subsequently introduced herbivores and pathogens attack introduced plants. As a result, introduced plant species can have a response to herbivory similar to native plants in as little as 150 years (Hawkes 2007; Schultheis et al. 2015). Constitutive chemical defences may therefore be maintained in the introduced range because of pressure from generalist herbivores, because good growing conditions ameliorate any trade-offs, or because the secondary compounds involved are maintained for functions other than herbivore defence.

One explanation for the similarity in chemical profiles is that some guilds of herbivores exert a similar pressure in the native and introduced ranges for these *Rumex* species. For example, in New Zealand, *Rumex* has re-associated with two generalist phloem-feeding herbivores from their native range in the early 1900’s (*Closterotomus norwegicus* (Gmelin), Miridae/Hemiptera) and in the 1960’s (*Philaenus spumarius* (L), Aphrophoridae/Hemiptera) respectively (Archibald et al. 1979; Myers and China 1928; Costan et al. 2022). The chemical defences tested in this study are effective against both chewing and sap-sucking insect herbivores including oxalates (Yoshihara et al. 1980; Yoshida et al. 1995), phenols (Leszczyǹski et al. 1985; Larsson et al. 1986) and tannins, which have both toxic and digestibility-reducing effects (Felton and Summers 1995; Harborne 2000; Barbehenn and Constabel 2011; Salminen and Karonen 2011). A similar pathogen load between regions could also result in selective pressure for similar defence profiles. Furthermore, in both their native and introduced ranges, *Rumex* plants are exposed to mammalian herbivores including deer (Cavers and Harper 1964), goats (Böhm and Finze 2003), sheep (Wilman et al. 1997; Zaller 2006), rabbits and hares and, to a lesser extent, cattle (Courtney and Johnston 1978), potentially causing greater damage than was observed by insect herbivores (Sakaoue et al. 1995; Costan et al. 2022). In temperate agricultural grasslands where *Rumex* plants typically grow, invertebrates, including introduced slugs (Wilson and Barker 2011), are often the dominant herbivores (Stein et al. 2010) and have been shown to feed on *Rumex* plants (Everwand et al. 2013). Slugs may be particularly important at the seedling stage (Hulme 1994). Thus, while in a previous study (Costan et al. 2022) we observed leaf and root damage of adult plants in the field to be significantly less in New Zealand, it is possible that non-insect herbivores and/or seedling herbivory is similar in both ranges and maintains chemical defences in the introduced range.

One alternative explanation for the maintenance of secondary metabolites in *Rumex* species is that the quantity of secondary metabolites may be constrained because these chemicals serve other functions in addition to their defensive role against herbivores. Oxalate is a common cellular constituent involved in Ca^2+^ regulation, ion balance, and metal detoxification (Morita et al. 2004; Brunner and Sperisen 2013). In New Zealand, many soils are acidic and have toxic levels of soluble aluminium, (Moir and Moot 2010; Whitley et al. 2018). The efflux of oxalate from the roots may act to chelate the Al^3+^ ions in the rhizosphere, forming stable and non-toxic complexes (Brunner and Sperisen 2013) and promoting plant growth. The physiological importance of oxalate in *Rumex* might therefore swamp any selection against it due to reduced specialist herbivore pressure. Similarly, polyphenols, including tannins, serve multiple roles, including protection against UV radiation (Haslam 1988; ChaichiSemsari et al. 2011; Hagerman and Buttler 1981; Hassanpour et al. 2011; Sis et al. 2011). High UV-B intensities can reduce seedling biomass, inhibit hypocotyl or root development and result in growth abnormalities (Dai and Upadhyaya 2002; Krizek 1975; Tevini et al. 1981; Tosserams et al. 1997). This may be particularly important for introduced *Rumex* plants as UV-B levels are higher in New Zealand than in Europe (McKenzie et al. 2007; Hock et al. 2019).

Selective pressure for reduced chemical defence production is generally hypothesized to result because of trade-offs in energy and resources for growth against defence, either as a result of direct resource limitations, or more commonly from plant allocation strategies that maintain fitness across a variable environment. In conditions of high resource availability, however, plants can express high levels of both growth and defence (Züst and Agrawal 2017). Given that the plants used in this experiment were grown under greenhouse conditions with ample water and nutrients and were not exposed to herbivores or high abiotic stresses, they might not have needed to reduce their investment in chemical defences. Furthermore, because plants sampled for chemical defences analysis were not exposed to herbivory, this study measured constitutive, not induced defences. However, our results were similar across both chemical analyses and the field assay, suggesting that induced defences also do not show a difference between provenances.

It is intriguing to note that the difference between the native and introduced provenances in the percentage of phenols that are easily oxidized tannins was significant for one of the three species (*R. conglomeratus*), as a result of greater decline in ellagitannins than in overall phenols. This suggests that a change in the herbivore community composition, or a shift in relative importance of different roles of the secondary metabolites resulted in a difference in the kinds of chemical defences produced. This could be because *R. conglomeratus* typically prefers wet environments, such as small streams, and therefore may be less exposed to mammalian herbivory than the other species, but given the small shift and lack of consistent results across species, this result, though significant, should be interpreted with caution.

## Conclusion

The results of this study provide evidence against the redirection of resources from chemical defences towards growth and reproduction a following the introduction of  three non-native *Rumex* spp. to New Zealand. Despite having previously shown that non-native *Rumex* plants experience less damage to leaf and root tissue (Costan et al. 2022), these same species did not respond by changing their defensive chemistry. This points to selection for these compounds by phloem feeders and/or molluscan seedling herbivores rather than chewing insects in the introduced range, limited flexibility in metabolic pathways as compounds play multiple functions and/or a lack of trade-offs between defence and growth. Whatever the cause of these patterns, they suggest that changes in plant chemistry linked to herbivore defence do not explain invasion success in *Rumex*.

## Data Availability

If the manuscript is accepted for publication, the data will be archived in Figshare.

## Code Availability

The code generated during the study is available upon reasonable request.
